# The Effect of Coach Gender on Competitive Weightlifting Performance for Men and Women Weightlifters

**DOI:** 10.3389/fsoc.2020.539566

**Published:** 2021-02-08

**Authors:** Abigail Mire, Elizabeth C. Heintz, Jeremy J. Foreman

**Affiliations:** School of Kinesiology, University of Louisiana at Lafayette, Lafayette, LA, United States

**Keywords:** age, conditioning, exercise, female, male, sex, strength, training

## Abstract

Gender of coaches relative to their athletes has recently garnered substantial attention in the public, the media, and academia. Relative to sports engulfed in controversy pertaining to men athletes being coached by women, such as professional baseball, basketball, and football, it is more common to see women coach men in competitive weightlifting, though only a small percent of men weightlifters are coached by women. In competitive weightlifting, coaches are responsible for both physically and mentally training athletes, and with the social barriers faced by women in a sport traditionally perceived as masculine, there may be mental training or communication benefits to training with a coach of a certain gender. Examining the gender of competitive weightlifters and their coaches, total weight lifted in the snatch and clean and jerk events are analyzed using OLS regression. Results indicate that men weightlifters perform better with men coaches. Women weightlifters perform better with men coaches until the age of 43, then they perform better with women coaches. The difference in performance may be due to several factors including historical bias against women in the sport.

## Introduction

Coaches are important and influential in sport, not only through their enhancement of athletes’ physical capabilities, but through their impact on athletes’ mental performances as well (Powers, 2008). Coaching has shifted from simply enhancing physical skills to promoting psychological improvement in conjunction with athletic performance (Jowett, 2005). The coach-athlete relationship is an important factor in an athlete’s determination and confidence, making the coach-athlete relationship the center of athlete development (Jowett, 2005). Therefore, it is important to have strength coaches from a variety of backgrounds to assist in all aspects of training and competition. While strength coaches come from a variety of backgrounds and have diverse experiences (Powers, 2008), there remains a gender imbalance among weightlifting coaches (O’Malley and Greenwood, 2018). However, little research has been conducted on the direct effects of strength and conditioning coaches on athletes, especially as it pertains to gender differences.

Allison and Butler (1984) stated that women athletes face psychological struggles, role conflicts, and identity crizes due to increased social barriers in sport compared to men. This is crucial for sports like weightlifting that are traditionally perceived as being masculine, potentially creating a greater sociological impact on women competitive weightlifters than men competitive weightlifters. Therefore, although Holloway and Baechle (1990) concluded that no evidence indicates that men and women should physically train differently, training programs should be tailored to the individual, including how coaches mentally prepare their athletes. Because mental state can impact physical performance and women have greater social challenges in sport than men, women likely experience greater psychological struggles than men that impact their performance, especially in a sport like competitive weightlifting (Abrahamsen et al., 2008; Judge et al., 2016; O’Malley and Greenwood, 2018). There is evidence that same-gendered coaches and athletes have better coach-athlete relationships (Jowett and Nezlek, 2012); therefore, it is likely that when women competitive weightlifters have women coaches whom experience similar social conflicts, women competitive weightlifters’ mental preparation could improve, thus improving physical performance as well. Given the recent and substantial attention given to the coach-athlete relationship (e.g., Jowett and Nezlek, 2012; Darvin et al., 2018), further analyses are needed to better understand how the gender of coaches may affect athlete performance.

Therefore, the purpose of this study is to investigate how coach gender impacts competitive weightlifting performance. Specifically, we aim to investigate whether competitive weightlifters perform better with coaches of the same or different gender. We hypothesize that competitive weightlifters with coaches of the same gender perform better than those with coaches of different genders. The findings from this study could impact weightlifting coaches and athletes, especially women participants, by determining if the gender of the coach affects athlete performance. Results from this study will also have implications for scholars who attempt to better understand psychological factors affecting women’s experiences within sport (e.g., Allison and Butler, 1984; O’Malley and Greenwood, 2018). Further, results will help confirm if there is a gender imbalance among competitive weightlifting coaches, which could affect the gender of coaches that athletic departments and other sport organizations hire.

## Literature Review

The objective of competitive weightlifting is to lift the more weight than other weightlifters within one’s respective weight class (Tobacyk et al., 2006). Because heavy loads are lifted during both practices and competitions, competitive weightlifters largely rely on their physical strength for both training and competition. However, evidence shows that mental strength can influence physical strength, and mental training increases performance more than physical strength training alone (Shackell and Standing, 2007). The goals of each participant are individualized and based on the potential of each lifter (Tobacyk et al., 2006); therefore, there are many different mental strategies used by coaches in attempts to improve powerlifting performance, including imagery, goal setting, and competition simulation (Shannon et al., 2012; Alexander et al., 2019).

Previous research also suggests that competitive trait anxiety is greater in athletes who compete in individual sports than athletes who compete in team sports (Judge et al., 2016). Competitive trait anxiety has been shown to significantly decrease powerlifting performance; therefore, psychological training should be included in powerlifting training programs (Judge et al., 2016). It is also possible that factors outside of competition, like social barriers and social support, affect athletes’ mental states and can impact their performance (Allison and Butler, 1984; Iso-Ahola, 1995). Given these findings, mental training and factors influencing the mental states of athletes are large influencers of athletic performance.

### Social Identity

Social identity serves as a basis for sport team formation and development, influencing appraisal and support within a team (Rees et al., 2015). However, athletes in individual sports (e.g., competitive weightlifting) face different challenges with social identity than athletes in team sports, particularly women (Joncheray et al., 2014). Rees et al. (2015) found that athletes may have different self and social identities in sport, having one identity as an athlete or teammate but using different identities to define themselves outside of a sport setting. This is especially applicable for women, as women athletes tend to have different personal and athletic identities, particularly in sports that are considered more masculine (Joncheray et al., 2014). For example, some professional women rugby players and boxers develop multiple identities to separate themselves from the social consequences of being considered masculine for playing rugby or boxing (Mennesson, 2000; Joncheray et al., 2014).

Social identity is also an important factor in sports leadership (Rees et al., 2015). Coaches who are able to implement athletes’ identities and beliefs in their coaching are more successful leaders in sport (Rees et al., 2015). Women coaches face similar social barriers as women athletes in sport, hindering their professional development and advancement (Norman and Rankin-Wright, 2016). Women coaches are the minority in sport, and thus face underrepresentation and oppression in sports leadership (Norman, 2010). Therefore, women coaches offer a unique perspective to women athletes who face similar struggles within sport.

Sport provides an environment for underrepresented populations, like women, to gain social acceptance and increase self-esteem (Melton and Cunningham, 2014). Even when faced with unjust treatment, underrepresented populations working in sport were able to have positive experiences and gain acceptance when they were supported by others in the same environment (Melton and Cunningham, 2014). Relatedly, support from women coach who face social barriers and barriers to acceptance in sport could be beneficial for women athletes who face similar social challenges, particularly in individual sports perceived to be masculine, like competitive weightlifting.

### Coach-Athlete Relationships

The relationship between coaches and their athletes has been shown to be a crucial aspect of an athlete’s development and success (Jowett, 2005). In recent decades, coaching roles have broadened to include mental preparation in conjunction with traditional physical training, expanding the importance of coaches to an athlete’s preparation for competition (Jowett, 2005). This shift has also provided opportunities for increased understanding of the coach-athlete relationship and how it impacts success (e.g., Jowett and Nezlek, 2012; Hampson and Jowett, 2014; Jowett, 2017; Darvin et al., 2018). Coaches provide the foundation for helping athletes increase mental factors related to physical performance, including determination and confidence (Jowett, 2005). Furthermore, successful coach-athlete relationships are significant predictors of team athletic success (Hampson and Jowett, 2014).

Given the increase in one-on-one interactions in individual sports, it is likely that coaches also have a large impact on athletes’ athletic performances, making the coach-athlete relationship a significant part of an athlete’s performance in a sport like weightlifting. Additionally, strong coach-athlete relationships lead to enhanced goal setting and achieving (Jowett, 2017), a factor of great importance in competitive weightlifting (Tobacyk et al., 2006). Finally, same-gendered coaches and athletes have higher quality relationships than opposite-gendered coaches and athletes (Jowett and Nezlek, 2012). Thus, it is likely that coach gender has an impact on performance, and more specifically in an individual sport, like competitive weightlifting, where coach-athlete interactions are increased.

### Gender Imbalances

Strength and conditioning coaches are responsible for improving athletic performance through physical and mental training (Powers, 2008). Recently, the presence and roles of strength and conditioning coaches have increased, especially within collegiate athletics (Powers, 2008); however, there is a significant gender imbalance among strength coaches, leaving women largely underrepresented as strength and conditioning coaches (Massey and Vincent, 2013; O’Malley and Greenwood, 2018). With sports and strength training historically being dominated by men, women strength coaches face barriers of traditional gender roles and societal disapproval (Holloway and Baechle, 1990; Magnusen and Rhea, 2009; O’Malley and Greenwood, 2018). Further, while it is common for men strength coaches to train both men and women athletes, women strength coaches typically work only with women athletes and rarely with men athletes (Magnusen and Rhea, 2009; Massey and Vincent, 2013).

Similar to strength coaches, gender imbalances are well-documented among weightlifting coaches as well. Because children are taught to view weightlifting as masculine, women weightlifters are perceived as more masculine and less feminine than non-weightlifters, leading to significantly fewer women powerlifting competitors than men (Jackson and Marsh, 1986; Holloway and Beachle, 1990). Women athletes face barriers of role conflicts and identity crizes due to societal expectations (Allison and Butler, 1984), especially in a sport like weightlifting that is often viewed as masculine. These social barriers can cause increased psychological stress for women athletes (Allison and Butler, 1984), which could lead to increased anxiety that decreases performance (Judge et al., 2016).

Such pressures from negative attitudes, biases, and stereotypes may hinder the athletic success of women. It is possible that having women strength coaches and role models who face similar societal struggles in sport can benefit women competitive weightlifters. Moreover, given the history of discrimination against women in sport, the pipeline for talented women coaches may be clogged, resulting in only the most talented of women weightlifting coaches being used (Madden, 2004). However, even the most talented women weightlifting coaches may not be able to overcome the social and psychological pressure faced in a sport perceived to be masculine and dominated by men. Therefore, we offer two competing hypotheses based on women’s competitive weightlifting performance and two competing hypotheses based on men’s competitive weightlifting performance:

1. Hypothesis 1a: Women competitive weightlifters with women coaches lift more weight in competition than with men coaches.
2. Hypothesis 1b: Women competitive weightlifters with men coaches lift more weight in competition than with women coaches.
3. Hypothesis 2a: Men competitive weightlifters with women coaches lift more weight in competition than with men coaches.
4. Hypothesis 2b: Men competitive weightlifters with men coaches lift more weight in competition than with women coaches.

## Methods

To examine the relationship between coach gender and competitive weightlifting performance, we use data from Olympic weightlifting competitions. Olympic-style weightlifting involves two events: 1) snatch and 2) clean and jerk. The snatch requires a weightlifter to bring the barbell from the floor to above the weightlifter’s head with arms completely extended upwards, in a continuous motion (Anton et al., 2004). The clean and jerk event is composed of two movements itself (i.e., the clean and the jerk). In the clean phase, the weightlifter raises the bar from the ground to the shoulders in a single movement and can pause before proceeding to the jerk phase (Anton et al., 2004). The jerk phase is the movement that requires the weightlifter to thrust the bar overhead, again extending the arms upward, and then completing the phase by repositioning the feet (Anton et al., 2004). After assessing the amount of weight lifted in the snatch and clean and jerk events, a total weight lifted figure is calculated as the sum of the two events.

Data were collected from the United States Weightlifting website at https://www.teamusa.org/USA-Weightlifting. The sample included 4,678 weightlifters absolute year-end rankings with the total weight lifted calculated for women and men weightlifters from the 2011 season. The 2011 season was selected because it was the only season where publicly available data with rankings and coach names were provided for men and women athletes on the United States Weightlifting website. However, 1,340 observations did not list a coach and another 53 observations had total weight lifted figures of 0 kg·s, and were excluded from the analysis, resulting in a sample of 3,285 usable observations.

To address the hypotheses pertaining to women and men weightlifters, two separate statistical analyses were conducted–one examining women weightlifters and one examining men weightlifters. The gender of the weightlifter was determined by whether the athlete was competing in the women’s or men’s weight classes.

The dependent variable in this study was the total amount of weight lifted in the snatch and clean and jerk events, measured in kgs (Total Weight Lifted). This variable was used to measure the performance of a weightlifter in 2011. Larger numbers are indicative of better performance, whereas smaller numbers (i.e., lower amounts of weight lifted) are indicative or worse performance.

The independent variable of interest was a dichotomous variable indicative of the coach’s gender (Woman Coach). Woman Coach was coded as a 1 if the weightlifter had a woman coach and a 0 if the weightlifter had a man coach. Woman Coach was used to examine the four hypotheses regarding the relationships between the genders of weightlifters and coaches and weightlifting performance.

The gender of the coach was largely based on coaches’ names listed in the 2011 absolute year-end rankings. Coach names were divided into three groups: those with traditionally man names, traditionally woman names, and gender-neutral names. For coaches with gender neutral names, internet searches were used to determine the gender of the coach. If the coaches were identified by an internet search, their pictures were used to classify them as men or women coaches and verified by two of the authors with over 90% consistency. There were 32 cases in which the coach was not identifiable by internet search. One additional case identified Master Splinter as the coach, which appeared to be a man, however, was excluded from the dataset due to apparently being a fictitious rat. Therefore, the sample size was reduced to 3,252 observations.

Given the relationship between age and weightlifting performance (Anton et al., 2004), three age-related independent variables were also included in the statistical analysis. The athletes age during the year of competition (i.e., 2011) was determined by subtracting birth year from 2011 (Age). Additionally, to better understand the effect of coach gender on the age-performance relationship, an interaction variable is included in the model that interacts Age with Woman Coach (Age × Woman Coach). Due to the curvilinear relationship previously found between age and weightlifting performance (Anton et al., 2004), the Age variable is squared to create an age squared variable (Age^2^).

Lastly, to control for the weight of the weightlifters, an ordinal variable that identified the weight class of the weightlifter was included in the analysis (Weight Class). Because there were seven weight classes for women and eight weight classes for men, Weight Class ranged from 0 to 6 in the analysis of women weightlifters and 0–8 in the analysis of men weightlifters. Weight classes for women ranged from the under 48 kg weight class to the above 75 kg weight class, whereas weight classes for men ranged from the under 56 kg weight class to the above 105 kg weight class.

Consistent with previous statistical analyses of weightlifting performance using a continuous dependent variable (e.g., Anton et al., 2004), we used ordinary least squares (OLS) regression to examine the relationships between gender, age, and weight class on total weight lifted in competitive Olympic-style weightlifting. We estimate the model with robust standard errors to account for heteroskedasticity. The regression estimation takes the following form:

$$\text{Total}\;\text{Weight}\;\text{Lifted}={\text{β}}_{\text{0}}+{\text{β}}_{1}\left(\text{Woman}\;\text{Coach}\right)+{\text{β}}_{2}\left(\text{Age}\right)+{\text{β}}_{3}\left(\text{Age}\times \text{Woman}\;\text{Coach}\right)+{\text{β}}_{4}\left({\text{Age}}^{2}\right)+{\text{β}}_{5}\left(\text{Weight}\;\text{Class}\right)+e$$

## Results

Table 1 reports the summary statistics of the mean, standard deviation, minimum, and maximum values for each variable. Total Weight Lifted for women ranges from 19 to 256 with a mean of 108.1 kg·s and for men ranges from 17 to 389 with a mean of 180.9 kg·s. Among women weightlifters, 12.3% have a woman coach, whereas 6.7% of men weightlifters have women coaches. Ages in the sample extend from 5 to 83 years old.

**TABLE 1:** Summary statistics.

Variable	Women weightlifters (N = 1,095)				Men weightlifters (N = 2,157)			
	Mean	Std. Dev.	Min	Max	Mean	Std. Dev.	Min	Max
Total weight lifted (kgs)	108.051	37.453	19	256	180.919	64.336	17	389
Woman coach (1 = yes, 0 = no)	0.123	0.329	0	1	0.067	0.250	0	1
Age	25.870	12.184	7	76	25.452	13.169	5	83
Weight class	3.029	1.878	0	6	3.666	2.062	0	7

Multivariate linear regression results for each independent variable are reported in Table 2 as well as the R^2^ values for each regression. The R^2^ for the analysis of the women weightlifters is 0.343 and the R^2^ for the analysis of men weightlifters is 0.483. Woman Coach has negative and statistically significant relationships with Total Weight Lifted. In this sample, women weightlifters with women coaches lift an average of about 22 kg·s less than women weightlifters with men coaches (*p* < 0.01). Similarly, men weightlifters with women coaches lift an average of about 16 kg·s less than men weightlifters with men coaches (*p* < 0.05). Therefore, Hypotheses 1b and 2b are supported by the analysis.

**TABLE 2:** Linear regression estimates for total weight lifted (kgs).

Variable	Women weightlifters		Men weightlifters	
	Coefficient	Std. Err.	Coefficient	Std. Err.
Woman coach	−22.142**	5.035	−16.172*	7.399
Age	5.032**	0.386	8.240**	0.403
Age × woman coach	0.518*	0.204	0.336	0.253
Age^2^	−0.079**	0.006	−0.115**	0.006
Weight class	5.746**	0.534	13.598**	0.553
Constant	26.061**	5.011	16.475**	5.305
R^2^	0.343		0.483	
Observations	1,095		2,157	

Note: *p < 0.05, two-tailed. **p < 0.01, two-tailed.

**FIGURE 1:**
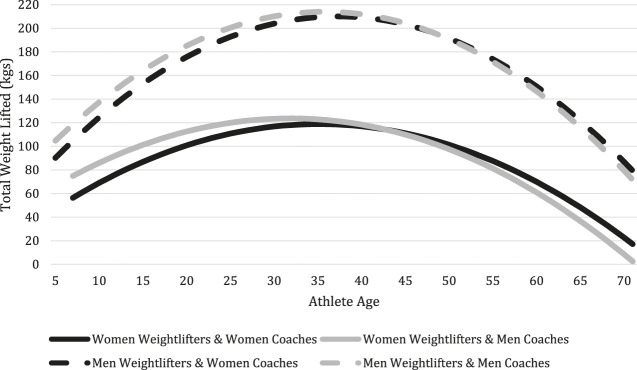
Predicted total weight lifted based on regression coefficient estimates, by age, athlete gender, and coach gender.

Also of interest, and related to coach gender, is the Age variable and its interaction with Woman Coach. For women weightlifters, each year increase in age is associated with an increase in the amount of weight lifted when working with a woman coach. More specifically, with each year of age, the average increase in weight lifted when a woman is coached by a woman is 0.52 kg·s (*p* < 0.05), however, there is no statistically significant effect for men weightlifters. Thus, there is support for Hypothesis 1a for more mature weightlifters. Additionally, the analysis indicates there is a curvilinear effect of age on total weight lifted for both women and men weightlifters. The estimated effects of coach gender and age are depicted in Figure 1.

## Discussion

The purpose of this study was to examine the effect of coach gender on both men and women weightlifting performance. We established that due to social and psychological pressures (Allison and Butler, 1984; Iso-Ahola, 1995), women coaches may perform worse, but could also perform better due to discrimination causing only the most talented coaches to remain in the labor market (e.g., Madden, 2004). Additionally, an added benefit of women weightlifters having women coaches may exist based on stronger female-female relationships, perhaps due to increased mutual support and understanding of their challenges (Jowett and Nezlek, 2012; Melton and Cunningham, 2014).

The regression analysis indicated that men weightlifters perform better with men coaches. Similarly, women weightlifters perform better with men coaches until the women weightlifters mature to about 43 years old, then they perform better with women coaches. While the evidence that men weightlifters perform better with men coaches refutes the idea that only the best coaches from underrepresented populations are gaining access to coaching positions and subsequently outperforming the majority (see Madden, 2004), it confirms the idea that either same gender coaches are optimal (Jowett and Nezlek, 2012) or that women coaches continue to experience social and psychological pressures that are detrimental to performance (Allison and Butler, 1984; Iso-Ahola, 1995). However, the results regarding the performance of women weightlifters with women coaches can lend additional insight into this phenomenon.

Given that women weightlifters perform better with men coaches prior to entering their 40 s, it appears that same gender coaches are not necessarily more or less beneficial for increasing weightlifting performance, despite Jowett and Nezlek (2012) finding that women athletes with women coaches reported having more satisfaction (e.g., with training) in their athlete-coach relationships. Interestingly, Jowett and Nezlek’s sample included only individual sport participants with a maximum age of 40. Thus, the discrepancy between the present study and Jowett and Nezlek’s findings may be based on the individual sport examined, biased perceptions in survey data, or a disconnect between relationship satisfaction and actual performance. For example, the sports examined by Jowett and Nezlek included cycling, golf, racquet sports, swimming, and track and field, where it is likely that women experience more acceptance (both within the sport and greater society) than in competitive weightlifting.

To demonstrate the disparities between women and men in competitive weightlifting, our findings show that only about 8.6% of weightlifting coaches in the sample were women. These results provide further evidence of the underrepresentation of woman leadership in sport (e.g., Norman, 2010), especially in competitive weightlifting. Additionally, women weightlifters make up a much smaller portion of the sample than men weightlifters in the sample (33.7%), showing the gender imbalance of both women weightlifters and coaches in weightlifting. As weightlifting is traditionally considered a masculine sport, these findings highlight the need for more encouragement and opportunities for women in the sport in both competitive and leadership roles.

While women are still trying to fight for acceptance within sport, men are typically taught to play sports or are encouraged to be involved in sports (Trolan, 2013); therefore, it is generally more accepted to find men in coaching positions in sports, especially sports traditionally perceived to be masculine, like weightlifting. Likewise, women coaches are marginalized within sport, often hindering their professional success (Norman, 2010; Norman and Rankin-Wright, 2016). Because of increased opportunities for men coaches, men weightlifting coaches may also have more experience than women coaches, thus further providing men with more and better opportunities. Interestingly, with a more equal representation of men and women coaches, Darvin et al. (2018) found that coach gender did not impact individual performance. With such a small representation of women coaches in the present study, it is possible that effects of coach gender on athlete success are overexaggerated. With increased women coaching representation, the negative effects of coach gender on younger women weightlifting performance may be negated or even reversed.

Nevertheless, more mature women weightlifters who have women coaches tend to have higher levels of performance. This finding could provide evidence of the impact of coach-athlete relationships on athlete success. If women experience more satisfaction with their women coaches in individual sports that have higher levels of women participation (Jowett and Nezlek, 2012), then as women weightlifters mature, they may be able to feel more comfortable with women coaches, despite competing in a sport vastly underrepresented by women.

Another possibility stemming from Jowett and Nezlek (2012) findings is that the women weightlifters may not reap the benefits of their relationships with women coaches until it has sufficiently developed and allowed for coaching improvements. If women experience better satisfaction in their training with women coaches, they are likely to be more forthcoming with feedback, which would allow women coaches to help the weightlifter improve her performance. Thus, the benefits realized from a strengthened bond overtime may not be immediately realized, but becomes more apparent as weightlifters mature and benefit from improved coaching.

## Conclusion

The results of this study indicate that women weightlifters performed better with men coaches until they reach their 40 s, then perform better with women coaches. However, men perform better with men coaches, regardless of age. While there are multiple reasons this could be occurring, our results also provide evidence of a significant gender imbalance among weightlifting coaches, with substantially fewer opportunities for women coaches. The findings of this study have implications for weightlifters, their coaches, and organizations that employ strength coaches (e.g., university athletic departments). Additionally, weightlifters and coaches can benefit from these results by understanding how age and coach gender can influence weightlifting performance. Scholars and coaches can also use the results from this study to further research and understand differences in men and women coaching strategies and weightlifters’ perceptions of having men or women coaches.

### Limitations

This research only analyzed data from 2011. Analyzing multiple years of data would allow for a more complete understanding of how coach gender impacts weightlifting performance. Further, data from more recent years could indicate whether coach gender still has similar effects on weightlifting performance or if women coaches have experienced more success after gaining more coaching experience. Additionally, the limitations of this quantitative analysis prevent us from better understanding the role of coach gender in competitive weightlifting; therefore, more qualitative methods (e.g., semi-structured interviews) are needed to fully understand the results from this study.

### Future Research

Future studies could include more control variables, like coaching experience and gym resources. Additionally, interviewing athletes could provide insight on weightlifter perceptions of men and women coaches (including cultural factors) and the impact of gender on coach-athlete relationships. Interviewing coaches could reveal differences in coaching tactics, personalities, and communication between men and women coaches and how these differences impact the performance of athletes in competition.

## Data Availability

The datasets generated for this study are available on request to the corresponding author.
